# Molecular Components of Nitrogen Fixation Gene Cluster and Associated Enzymatic Activities of Non-Heterocystous Thermophilic Cyanobacterium *Thermoleptolyngbya* sp.

**DOI:** 10.3390/life11070640

**Published:** 2021-06-30

**Authors:** Meijin Li, Lei Cheng, Jie Tang, Maurycy Daroch

**Affiliations:** 1 School of Environment and Energy, Peking University Shenzhen Graduate School, 2199 Lishui Rd., Shenzhen 518055, China; 1801213472@pku.edu.cn; 2 Beijing Engineering and Technology Research Center of Food Additives, Beijing Technology & Business University, Beijing 100048, China; chenglei@btbu.edu.cn; 3 School of Food and Bioengineering, Chengdu University, Chengdu 610106, China; tangjie@cdu.edu.cn

**Keywords:** biological N_2_ fixation, Nif genes, gene cluster, genome analysis, *Thermoleptolyngbya*, non-heterocystous cyanobacteria

## Abstract

*Thermoleptolyngbya* is a genus of non-heterocystous cyanobacteria that are typical inhabitants of hot spring microbial mats. These filamentous cyanobacteria are capable of nitrogen fixation. In this study, we examined the genome sequences of five publicly available *Thermoleptolyngbya* strains to explore their nitrogen fixation gene cluster. Analysis of the nitrogen-fixation clusters in these extremophilic strains revealed that the cluster is located in a single locus in *Thermoleptolyngbyace*. The average nucleotide and amino acid identities of the nitrogen-fixation cluster combined with phylogenetic reconstructions support that nitrogen fixation genes in *Thermoleptolyngbyaceae* are closely related to one another but also heterogeneous within the genus. The strains from Asia, and China more specifically, generate a separate clade within the genus. Among these strains *Thermoleptolyngbya* sp. PKUAC-SCTB121 has been selected for experimental validation of clade’s nitrogen fixation capacity. The acetylene reduction experiments of that strain shown that the strain can reduce acetylene to ethylene, indicating a fully functional nitrogenase. The activity of nitrogenase has been tested using different gas compositions across 72 h and exhibited a two-phase trend, high nitrogenase activity at the beginning of the assay that slowed down in the second phase of the analysis.

## 1. Introduction

Biological N_2_ fixation (BNF) is the process in which atmospheric N_2_ gas is converted into ammonia nitrogen by a nitrogen-fixing microorganism. The nitrogen fixation is driven by a cluster of *nif* genes products that catalyze this important reaction [1]. The nitrogenase complex consists of two metalloprotein components of the molybdenum iron (MoFe) protein and iron (Fe) protein [2]. For the microorganisms that could perform BNF, cyanobacteria are the most diverse photosynthetic bacteria, widely distributed in various conditions, and play an essential role in the BNF.

A hot spring is a place where warm or hot groundwater springs from the Earth’s crust on a regular basis for at least a predictable part of the year and is significantly above the average temperature. The water emanating from a hot spring is heated by geothermal heat. A previous study showed that genomes and metagenomes of cyanobacteria that form hot spring communities harbor a conserved *nif* gene cluster, suggesting that these strains have a potential to perform nitrogen fixation [3]. The role of individual microbes in nitrogen cycling as environmental conditions vary over space and time. The present upper-temperature limit for N_2_ fixation (92 °C) was reported in an organism isolated from a marine hydrothermal vent. The upper-temperature limit for N_2_ fixation in terrestrial environments is 82 °C [4].

Thermophilic cyanobacteria isolated from hot springs are increasingly studied due to their environmental and biotechnological importance [5,6]. Cyanobacteria exhibit remarkable diversity in their morphological structures and can be broadly divided into heterocystous and non-heterocystous based on their mode of nitrogen fixation. The N_2_-fixing system of non-heterocystous cyanobacteria markedly differs from that of heterocystous counterparts. Heterocystous filamentous cyanobacteria, such as *Fischerella thermalis*, have attracted early attention for their role in the cycling of nitrogen in thermal ecosystems [7]. Their mode of action is relatively well described [8]. The nitrogen-fixing capacity of non-heterocystous cyanobacteria in thermal ecosystems is less studied.

To some extent, the mechanisms of non-heterocystous cyanobacteria are more diverse [9]. This is especially important considering that non-heterocystous cyanobacteria cannot compartmentalize nitrogen fixation; therefore, an alternative mechanism to avoid the nitrogenase inhibition with oxygen need to be implemented in non-heterocystous strains [2,10]. Previous studies showed that there are various strategies to protect the enzyme complex against oxygen. For example, *Trichodesmium* sp., a marine filamentous cyanobacterium, exhibits exceptional ability to fix atmospheric nitrogen during the day. Whilst the strain does not differentiate into heterocysts, the localization of its Fe-protein of nitrogenase (dinitrogenase reductase) is confined to a limited subset of cells (on average 14%) in the sections randomly distributed across the filaments. The nitrogenase containing cells perform the O_2_ protective function, but they are not *bona fide* heterocysts [11,12].

Recently a new genus of filamentous cyanobacteria, *Thermoleptolyngbya* was delineated from the polyphyletic *Leptolyngbya,* and several species have been proposed within this genus [13,14]. Strains of this genus are non-heterocystous, and they could play an essential role in the nitrogen cycle within thermal ecosystems, as recently suggested by a study on *Thermoleptolyngbya* sp. O-77, collected from hot springs of Kumamoto, Japan [15]. Thanks to the advances of modern DNA sequencing technology and genomics, analysis of the genetic information of both cyanobacterial isolates and genome assemblies extracted from metagenomic bins created during hot spring community sequencing is now feasible. The increased sequence pool allows for a better understanding of nitrogen fixation in the thermal niches [16]. Simultaneously, as new cyanobacterial strains are isolated from thermal sites worldwide [17], and their genetic modification systems are being developed [18,19], experimental validation of sequence data is also increasingly possible. Interestingly, complete nitrogen fixation gene clusters have been identified in the genomes of *Thermoleptolyngbya*, which could be particularly interesting considering their ability to thrive in thermal environments. It also suggests that they are capable of providing nitrogen to the microbial mats they are inhabiting. These advances make *Thermoleptolyngbya* genus potentially interesting for studying nitrogen fixation among thermophilic non-heterocystous cyanobacteria and complement the work done on heterocystous thermal strains belonging to *Fisherella*.

In this study, we used the whole genome sequence of five *Thermoleptolyngbya* strains deposited in the public databases; compared and assessed their nitrogen-fixation gene cluster information. To complement bioinformatic analysis, we have selected an isolate *Thermoleptolyngbya* PKUAC-SCTB121, isolated from hot springs of Ganzi prefecture, Sichuan, China, from our culture collection as a representative of the genus and assessed its acetylene reduction capacity to provide experimental evidence of their nitrogen-fixing capability.

## 2. Materials and Methods

### 2.1. Genomic Sequence Information

All the sequence data used in this study were downloaded from NCBI Database. Table 1 shows their Genebank numbers (Table 1). Strains PKUAC-SCTB121 and PKUAC-SCTA183 (hereafter B121 and A183, respectively) were sequenced, assembled and annotated by our group using a hybrid sequencing approach [14].

### 2.2. Nitrogen Fixation Gene Cluster, ANI and AAI Analysis

The nitrogen fixation gene cluster containing 27 well-defined genes and 2 unknown hypothetical proteins was extracted from the B121 genome combined with the results published elsewhere [15] and the annotation data of strains used in this study. The 29 genes were mapped against four other *Thermoleptolyngbya* sp. strains using Geneious mapper (Biomatters, Melbourne, Australia) on default settings. All genes of the nitrogen fixation cluster were extracted, then 25 genes of each strain were concatenated as one string. The Average Nucleotide Identity (ANI) and Average Amino Acid Identity (AAI) analyses between these five concatenated strings were performed by an online tool devised by Kostas lab (Available at http://enve-omics.ce.gatech.edu/ accessed on 15 June 2021 [20]).

### 2.3. Phylogenetic Analysis and KEGG Pathway

MEGA-X software 10.1.7 (Available at https://www.megasoftware.net/ accessed on 17 June 2021) [21] was used to perform phylogenetic analyses of *nif*H and 16S rRNA genes. A total of 19, both heterocystous and non-heterocystous cyanobacteria, were assessed for the relationships of their *nif*H genes using the Maximum Likelihood method, parameters were set according to Tang [22]. 16S rRNA of 18 heterocystous and non-heterocystous cyanobacteria strains was used to perform a phylogenetic analysis to indicate the evolutionary relationship. Metagenomic bins belonging to strains *Thermoleptolyngbya* sp. C42_A2020 from Chile and *Thermoleptolyngbya* sp. M55_K2018 from India were excluded from the 16S analysis due to lack of sequence availability. Concatenated 25 genes of nitrogen gene cluster were used to perform MLSA (Multi Locus Sequence Analysis). The predicted gene-coding sequences were submitted to KEGG database, and the resultant metabolic pathway of nitrogen-fixation was analyzed.

### 2.4. B121 Cultivation and Maintenance

**Figure 1:**
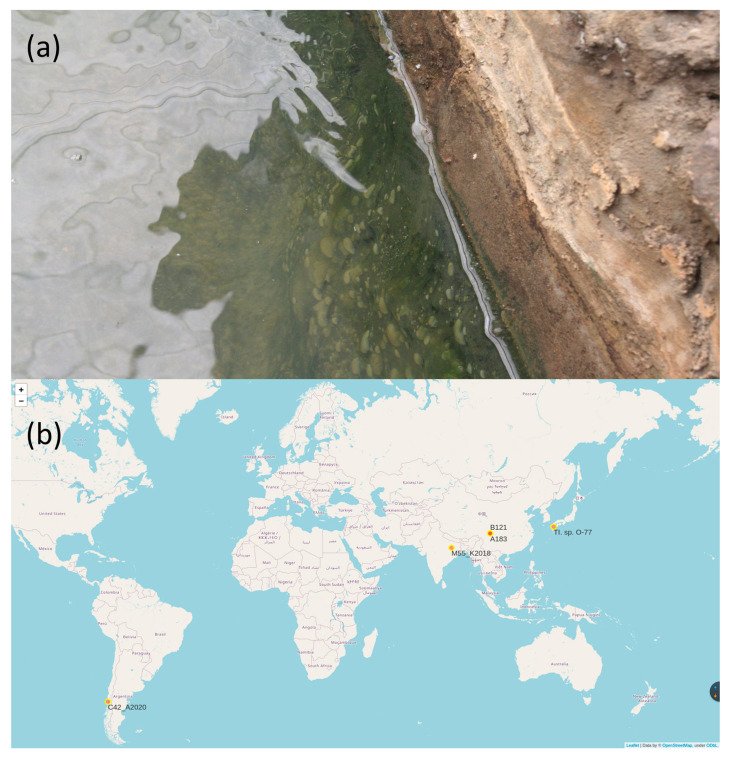
(**a**) Presents the sampling site of B121 strain, and a microbial mat in the pond from which the strain was isolated; (**b**) the geographical distribution of five strains analysed in this study.

The strain B121 used in the experiment was collected from the hot spring in Ganzi Prefecture [17]. The GPS location is 30°15′57″ N, 101°52′24″ E (Figure 1); and the temperature range is 53.1 °C; the environmental pH range is 6.35 [5,17]. The strain was cultured with BG-11 medium [23] and stored in a constant temperature light incubator. The relevant conditions of the incubator are set: temperature is 45 °C, light intensity is 2000 lux, photoperiod 16L:8D.

### 2.5. B121 Electron Microscopy

Electron microscopy has been performed essentially as described elsewhere [14]. Briefly, after harvesting, the cells were sequentially fixed using SEM fixative (Servicebio, Wuhan, China), post-fixed with 0.1M phosphate buffer (pH 7.4), dehydrated with a gradient of ethanol, dried with Critical Point Dryer, and coated with a conductive metal coating. Cells were observed under a scanning electron microscope (SEM) (HITACHI, SU8100). SEM micrographs of strain B121 were obtained with 3000× magnification. For transmission electron microscopy (TEM) cells were fixed in TEM fixative (Servicebio, Wuhan, China), post-fixed, and dehydrated, and embedded in resin. Finally, the embedded cells were cut 60–80 nm thin slices using an ultra-microtome and stained. Cells were observed under a transmission electron microscope (TEM) (HT7800/HT7700, Hitachi, Tokyo, Japan) to obtain images of strain B121 with a 7000× magnification (Wuhan Service Biotechnology Co. Ltd., Wuhan, China).

### 2.6. B121 Nitrogenase Assay

Cells of B121 pre-cultured in the nitrate-containing, standard BG-11 medium, were washed twice with nitrate-free medium (Stock 1 of BG-11 medium was replaced by ultra-pure water of equal volume). The washed cells were used under all experimental conditions. Nitrogenase assay methodology was adapted from Chen et al. [24]. Under nitrate-free conditions, cells resuspended in nitrate-free BG-11 were grown under three different N_2_ concentrations in inert gas mixtures: (Ar/N_2_/CO_2_ = 79/20/1 [*v/v/v*%]), (Ar/N_2_/CO_2_ = 90/9/1 [*v/v/v*%]), and (Ar/CO_2_ = 99/1 [*v/v*%]). The cylindrical vials of the total volume of 60 mL were filled with 20 mL of culture to provide sufficient headspace for sampling; flushed for 5 min with each of the different gas mixtures; and subsequently tightly sealed with rubber septum seals. Cell cultures were grown under the 12L:12D photoperiod under the illumination of 30 μmol m^−2^ s^−1^ at 45 °C, without shaking. Samples were collected and used to measure acetylene reduction in 12 h time intervals during the culture period of 72 h. The assay was initiated using an injection of 2 mL of acetylene gas, and nitrogenase activity was assessed based on the acetylene reduction reaction. Gaseous products were collected into the air pockets and sealed immediately. Air pockets were collected, and ethylene concentration was measured using a gas chromatography-hydrogen ion flame detector/mass spectrometry detector GC2014C (Shimadzu, Kyoto, Japan) by Qingdao BYNE Testing Technology Services Co., Ltd.

## 3. Results

### 3.1. Nitrogen Fixation Gene Cluster, ANI and AAI Analysis

#### 3.1.1. Nitrogen Fixation Gene Cluster

**Figure 2:**
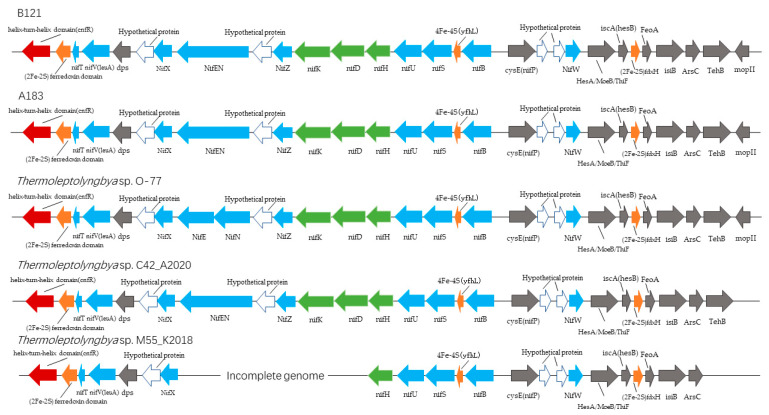
Nitrogen fixation gene clusters of *Thermoleptolyngbya* strains.

The result showed that B121, A183, *Thermoleptolyngbya* sp. O-77, and *Thermoleptolyngbya* sp. C42_A2020 contained 29 conserved genes involved in the nitrogen fixation gene cluster (Supplementary Materials Figure S1, Table 2), as well as hypothetical proteins between those 29 genes. The *nif* genes of the FeMo cofactor synthesis and maturation components were identified in the genome sequences of these four strains. The *nif* gene cluster was divided into two regions with different transcriptional directions in all strains analysed, showing significant consistency of that genomic fragment (Supplementary Materials Figure S1). The *nifHDK* genes were located centrally in the nitrogen fixation gene cluster (Figure 2). The fifth strain analysed, *Thermoleptolyngbya* sp. M55_K2018 had only 19 nitrogen fixation genes, lacking all-important: *nifK*, *nifD*, *nifEN*, *nifZ*, *mopII*, and tellurite resistance protein *TehB*, genes. The detailed analysis of the sequences suggests that this metagenome-assembled draft genome is incomplete and should be completed before final conclusions could be made. The KEGG analysis of the obtained data suggests that except for the incomplete M55 genome, all other strains exhibited a fully functional nitrogen fixation pathway (Supplementary Materials Figures S2–S5).

#### 3.1.2. ANI and AAI Analysis

Average Nucleotide (ANI, Table 3) and Amino Acid (AAI, Table 4) of concatenated sequences of the 25-long gene cluster suggest that nitrogen-fixation cluster in *Thermoleptolyngbya* strains is conserved. The two Sichuan strains show the highest identity at both nucleotide and amino acid level, followed by Japanese strain O-77. More geographically distant strains show lower similarities. Interestingly, whilst at the nucleotide level, the Chinese and Japanese strains of *Thermoleptolyngbya* are near identical, there is a greater divergence at the amino acid level, suggesting a selection pressure for amino acid change between these strains.

### 3.2. Phylogenetic Analysis

**Figure 3:**
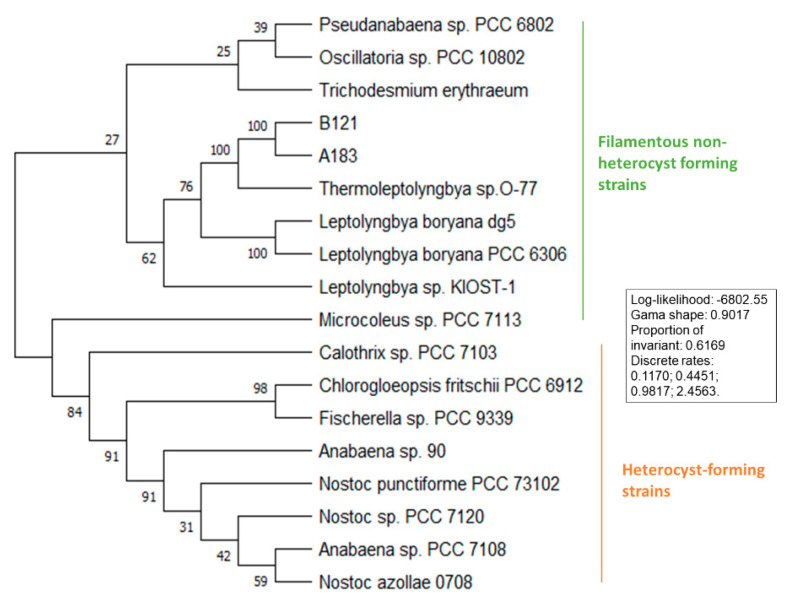
Phylogenetic tree of 16S rRNA gene.

**Figure 4:**
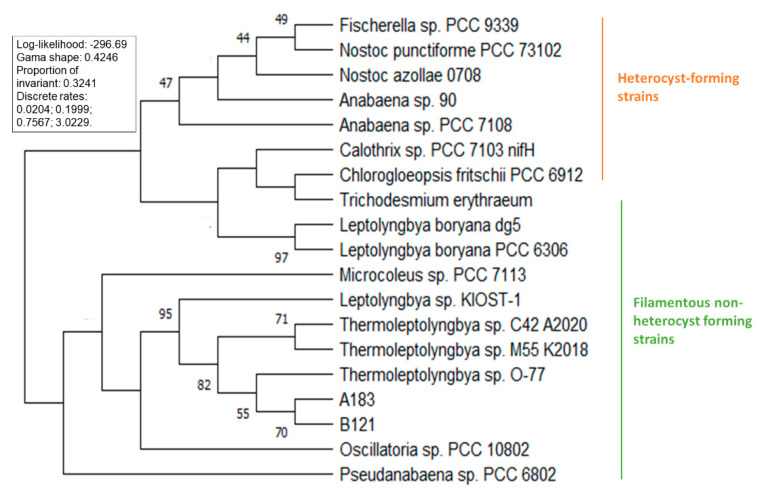
Phylogenetic tree of nifH gene.

Several phylogenetic trees have been constructed to position *Thermoleptolyngbya* strains among other nitrogen-fixing cyanobacteria. Analysis of the 16S phylogeny separates the strains into three well-defined clades of filamentous heterocystous and non-heterocystous strains, with unicellular cyanobacteria forming an outgroup. Among filamentous non-heterocyst forming strains, the strains separate into *Leptolyngbya* (*Leptolyngbyaceae*) and *Thermoleptolybya* (*Ocullatelaceae*). Within the *Thermoleptolybya* clade, the two strains isolated from Ganzi prefecture of Sichuan province separate from the Japan-originated O-77 (Figure 3). The heterocyst-forming strains follow the previously published distribution [25]. Phylogenetic analysis of the nifH gene for 19 strains presented in Figure 4 showed that all five strains of *Thermoleptolybya* formed a well-defined clade along with other non-heterocystous cyanobacteria. Interestingly, strains that belong to the *Leptolyngbya* clade separated into two distinct clades showing higher divergence.

**Figure 5:**
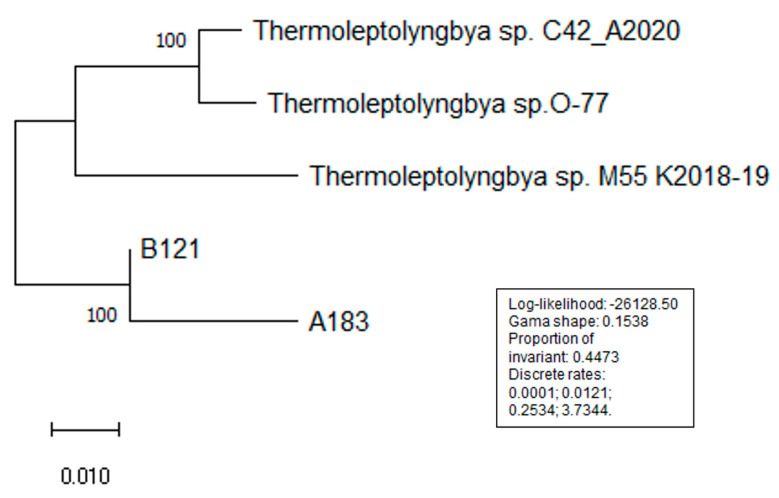
MLSA analysis for 25 genes of nitrogen fixation gene cluster.

Finally, the MLSA phylogenetic tree (Figure 5) also showed these four strains clustered well whilst maintaining divergence of Sichuan strains from the strains isolated from Japan (O 77), Chile (C42) and India (M55). Thermoleptolyngbya sp. M55_K2018 lacked six genes, including essential nifD and nifK. These genes were therefore excluded from this analysis throughout the dataset to maintain the maximal strain number for the analysis.

### 3.3. Morphological Investigation of B121

**Figure 6:**
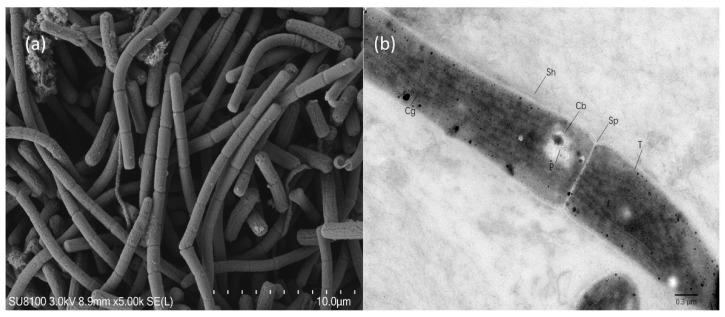
(**a**) SEM picture showing cells of B121 under acetylene reduction condition; (**b**) TEM picture of B121 (Cb carboxysome, Cg cyanophycin granule, P polyphosphate body, Sh sheath, Sp septum, T thylakoid).

Figure 6 presents the cells of *Thermoleptolybya* sp. B121 grown under nitrogen fixation conditions. It can be seen from the figure that the B121 did not differentiate into heterocysts, supporting the earlier claims that the B121 is a non-heterocytous cyanobacterium. Besides, according to SEM pictures, the appearance of B121 under nitrogen-fixation conditions did not differ from the strain grown under nitrate-replete conditions, which also shows that the nitrogen fixation capacity had little effect on the cell morphology. In addition, TEM picture showed a cyanophycin granule, which is associated with nitrogen storage, one of the mechanisms of how B121 cells store nitrogen; the relevant synthase has also been identified in the organism’s genome.

### 3.4. Nitrogenase Activity of Cell Suspensions of Thermoleptolyngbya sp. B121 Cells

**Figure 7:**
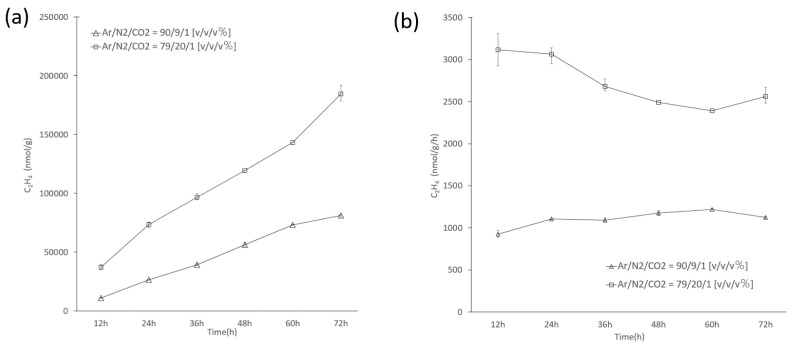
Acetylene reduction assay performed by cultures of *Thermoleptolyngbya* sp. B121 grown under different nitrogen concentrations (**a**) Cumulative ethylene production (nmol/g_biomass_) of cultures of *Thermoleptolyngbya* sp. B121; (**b**) Average ethylene production (nmol/g_biomass_/h) of the strain *Thermoleptolyngbya* sp. B121 per unit time of cultivation.

Considering that based on comparative genomic analysis, *Thermoleptolyngbya* strains form distinct clades using 16S, *nifH* and MLSA methodology *Thermoleptolyngbya* sp. B121 was selected as a representative strain for further study and assessment of its nitrogen fixation ability. To study the nitrogen fixation capacity of the cell suspensions the acetylene reduction activity test was used. The experiment was performed at the temperature of 45 °C and light intensity of 30 μmol m^−2^ s^−1^, both physiological to that strain [17]. Two nitrogen concentrations were tested, 9 and 20% (*v*/*v*), and the strain’s ability to reduce acetylene to ethylene was analyzed in the course of 72 h. Throughout the experiment, the amount of acetylene that has been reduced to ethylene has been steadily increasing (Figure 7a) and its amount was proportional to the concentration of nitrogen in the gas mixture. Moreover, a steady increase of ethylene content was observed regardless of the lighting conditions suggesting that cells were unlikely to photosynthesize during the assay. After 72 h, the ethylene content reached 81,159 nmol/g for the lower concentration of nitrogen. When the gas composition was Ar /N_2_/CO_2_ = 79/20/1 [*v/v/v*%], the amount of acetylene being reduced to ethylene increased. At the 72th hour, the ethylene content reached 184,618 nmol/g. Figure 6b shows that nitrogen concentration in the gas phase had a significant impact on the acetylene reduction assay. A direct correlation between the availability of nitrogen and detected in cellular suspensions enzymatic activity of the nitrogenase assessed with a proxy assay employing acetylene reduction could be observed. Analysis of the time course nitrogenase cellular suspension activity (Figure 7b) revealed that enzymatic activity assessed with acetylene reduction assay remains relatively stable at values of approximately 1000 nmol/g/h for a lower concentration of the nitrogen gas Ar/N_2_/CO_2_ = 90/9/1 [*v/v/v*%]. Meanwhile, at higher concentration, i.e., Ar/N2/CO2 = 79/20/1 [*v/v/v*%], the enzymatic activity starts from much higher values and subsequently stabilizes after 48 h of the assay by dropping by approximately 25% to 2400 nmol/g/h. The initial concentration of N_2_ in the gaseous mixture is likely therefore to have an impact on detected nitrogenase activity of cellular suspensions and probably on the expression and/or activation of the nitrogen fixation pathway.

## 4. Discussion

The results have shown that the nitrogen-fixation cluster, situated in a single genomic locus, including key enzymes of *nif* gene operon in *Thermoleptolyngbya* strains, is relatively conserved within the genus. Although strain *Thermoleptolyngbya* sp. M55_K2018 lacks six of the essential genes, the identity of its remaining genes of the cluster follows other strains of the genus. It is reasonable to analyze them again when the complete genome of *Thermoleptolyngbya* sp. M55_K2018 becomes available. Simultaneously its components are similar to other nitrogen-fixing organisms that operate *nif* operon [10,25].

Analysis of the nitrogenase activity of the selected B121 strain revealed that the strain is likely to possess a fully functional nitrogenase system based on its ability to reduce acetylene to ethylene. Interestingly activity of the enzyme depends on the initial concentration of the molecular nitrogen in the reaction mixture. These findings are consistent with previous findings on *Thermoleptolyngbya* sp. O-77 [15], where nitrogenase activities at 9% nitrogen and 20% nitrogen were markedly different. Analysis of the time course of nitrogenase activity throughout the 72 h period is different from that of the O-77 strain. The activity of the nitrogen-fixing enzyme shows relatively stable activity throughout an entire experiment and fails to follow the circadian pattern of the O-77 strain [15]. Similar results have been found in other cyanobacteria, namely *Symploca* PCC 8002 that exhibited the ability of nitrogen fixation under photosynthetic conditions through compartmentalization of nitrogenases in only a part of the cells forming a filament [26]. Meantime, the filamentous non-heterocystous cyanobacterium *Schizothrix*, isolated from a hot spring in Spain, showed that its N_2_ fixation in the light was significantly increased by an inhibitor of PSII and oxygen evolution, DCMU (3-(3,4-dichlorophenyl)-1,1-dimethylurea), and anaerobic conditions while no nitrogenase activity was found in the dark [27]. Conversely, studies of the non-heterocystous cyanobacterium, *Leptolyngbya nodulosa* shown through acetylene reduction assays that these cultures fixed nitrogen in the dark period of a diurnal cycle [28]. Therefore, it would be interesting to explore if a similar mechanism is typical for *Thermoleptolyngbya* sp. Similarly, there were earlier reports of aerobic nitrogenase activity shown by *Trichodesmium* NIBB1067. Even at a partial pressure of oxygen (Po_2_) of ~3 atm, the acetylene reduction by *Trichodesmium* sp. remained at half of the maximum, similarly to results obtained for heterocystous cyanobacteria. [29]. Additionally, nitrogenase of *Leptolyngbya nodulosa* could also be active under micro-oxygenic conditions [28]. An alternative explanation of the phenomenon could be the cohabitation of heterotrophic bacterium that could consume the oxygen from photosynthetic culture and creating a micro-oxygenic environment to allow uninterrupted nitrogen fixation by the hot spring cyanobacterium. Further studies are required to explore the non-circadian character of nitrogenase activity in *Thermoleptolyngbya* strains of Ganzi.

## 5. Conclusions

All five genome sequences of *Thermoleptolyngbya* shared a common nitrogen fixation gene cluster reinforcing the claim that the genus belongs to thermophilic non-heterocystous nitrogen-fixing cyanobacteria. Analyses of the gene cluster sequences revealed that strains are likely to possess a fully functional nitrogen-fixation gene cluster. Moreover, all five strains show a close evolutionary relationship between one another and cluster together on common phylogenetic tree branches. Within the *Thermoleptolyngbya* genus, the strains isolated from Sichuan province of China form a separate group using both standard taxonomic markers and their nitrogen fixation gene cluster. Experimental validation of the nitrogen-fixing capacity further reinforces the claim about the functionality of the pathway. The results have shown that *Thermoleptolyngbya sp.* strain is capable of nitrogen fixation at elevated temperatures characteristic of hot spring ecosystems. Finally, the non-circadian pattern of nitrogenase activity in this strain should be explored in the future.

## Figures and Tables

**Table 1:** Origin and accession numbers of *Thermoleptolyngbya* sp. genomes.

Strain	Accession Number	Genome Description	Strain Origin
*Thermoleptolyngbya* sp. PKUAC-SCTB121	CP070366.1	Complete genome sequence	30°15′57″ N 101°56′55″ E
*Thermoleptolyngbya* sp. PKUAC-SCTA83	CP053661	Complete genome sequence	30°05′14″ N 101°52′24″ E
*Thermoleptolyngbya* sp. O-77	AP017367	Complete genome sequence	32°48′11″N 130°42′28″E
*Thermoleptolyngbya* sp. C42_A2020	JACYLP000000000.1	Metagenomic bin	29°69′00″N 83°68′00″E
*Thermoleptolyngbya* sp. M55_K2018	DVEA00000000.1	Metagenomic bin	39°51′00″S 72°50′00″W

**Table 2:** The 29 genes of nitrogen fixation cluster in *Thermoleptolyngbya*.

Gene Name (B121 Convention)	Length (bp)	Function or Putative Function
helix-turn-helix domain (cnfR)	1599	Transcriptional regulator
(2Fe-2S) ferredoxin domain	654	(2Fe-2S) ferredoxin domain-containing protein
nifT	204	putative nitrogen fixation protein
NifV(leuA)	1122	Homocitrate or 2-isopropylmalate synthase
dps	543	DNA starvation/stationary phase protection protein
Hypothetical protein	477	NifX-associated protein
NifX	426	Nitrogen fixation protein
NifEN	2760	bifunctional nitrogenase iron-molybdenum cofactor biosynthesis protein NifEN
Hypothetical protein	306	Nitrogenase C-terminal domain-containing protein
NifZ	303	nitrogen fixation protein
nifK	1578	Nitrogenase molybdenum-iron protein beta chain
nifD	1455	nitrogenase molybdenum-iron protein alpha chain
nifH	876	Nitrogenase iron protein
nifU	906	Fe-S cluster assembly protein
nifS	1194	Cysteine desulfurase
4Fe-4S (yfhL)	375	4Fe-4S binding protein
nifB	1491	nitrogenase cofactor biosynthesis protein NifB
cysE (nifP)	726	serine O-acetyltransferase
Hypothetical protein	267	unknown
Hypothetical protein	219	unknown
NifW	318	nitrogenase-stabilizing/protective protein
HesA/MoeB/ThiF	783	Molybdopterin-synthase adenylyltransferase
iscA (hesB)	369	iron-sulfur cluster assembly accessory protein
(2Fe-2S) fdxH	303	2Fe-2S iron-sulfur cluster binding domain-containing protein
FeoA	312	ferrous iron transport protein A
isiB	531	Flavodoxin
ArsC	414	nitrogenase-associated protein
TehB	603	tellurite resistance protein
mopII	210	Molybdenum-pterin-binding protein 2

**Table 3:** ANI analysis for concatenated 25 genes for five strains.

ANI Identity	B121	A183	O-77	C42_A2020	M55_K2018
B121		99.95	93.97	93.84	95.54
A183	99.95		93.87	94.02	95.23
O-77	93.97	93.87		98.10	94.96
C42_A2020	93.84	94.02	98.10		95.23
M55_K2018	95.54	95.23	94.96	95.23	

**Table 4:** AAI analysis for concatenated 25 genes for five strains.

AAI Identity	B121	A183	O-77	C42_A2020	M55_K2018
B121		99.90	95.95	96.87	96.64
A183	99.90		96.18	96.69	97.36
O-77	95.95	96.18		98.77	95.98
C42_A2020	96.87	96.69	98.77		96.92
M55_K2018	96.64	97.36	95.98	96.92	

## Data Availability

Publicly available datasets were analyzed in this study. This data can be found here: CP070366; CP053661; AP017367; JACYLP000000000.1; DVEA00000000.1.

## Supplementary Materials

The following are available online at https://www.mdpi.com/article/10.3390/life11070640/s1, Figure S1: Gene cluster of strains in this study, Figures S2–S5: KEGG pathway of nitrogen metabolism for strain B121, A183, *Thermoleptolyngbya* sp. O-77, *Thermoleptolyngbya* sp. C42_A2020.
