# Supplementary material for: Molecular Components of Nitrogen Fixation Gene Cluster and Associated Enzymatic Activities of Non-Heterocystous Thermophilic Cyanobacterium Thermoleptolyngbya sp
Source: Life (Basel). 2021 Jun 30;11(7):640. doi: 10.3390/life11070640 (PMC8307165; doi:10.3390/life11070640)
Supplement: Supplementary file 1 [file life-11-00640-s001.zip › life-1264173-supplementary.pdf]

life

MDPI

Supplementary Materials:

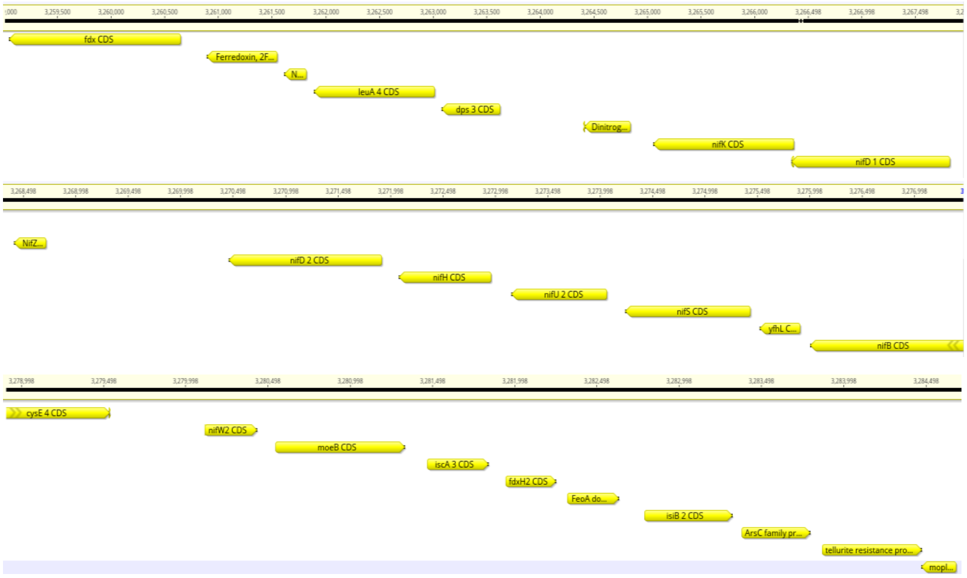

Figure.S1 Gene cluster of strains in this study

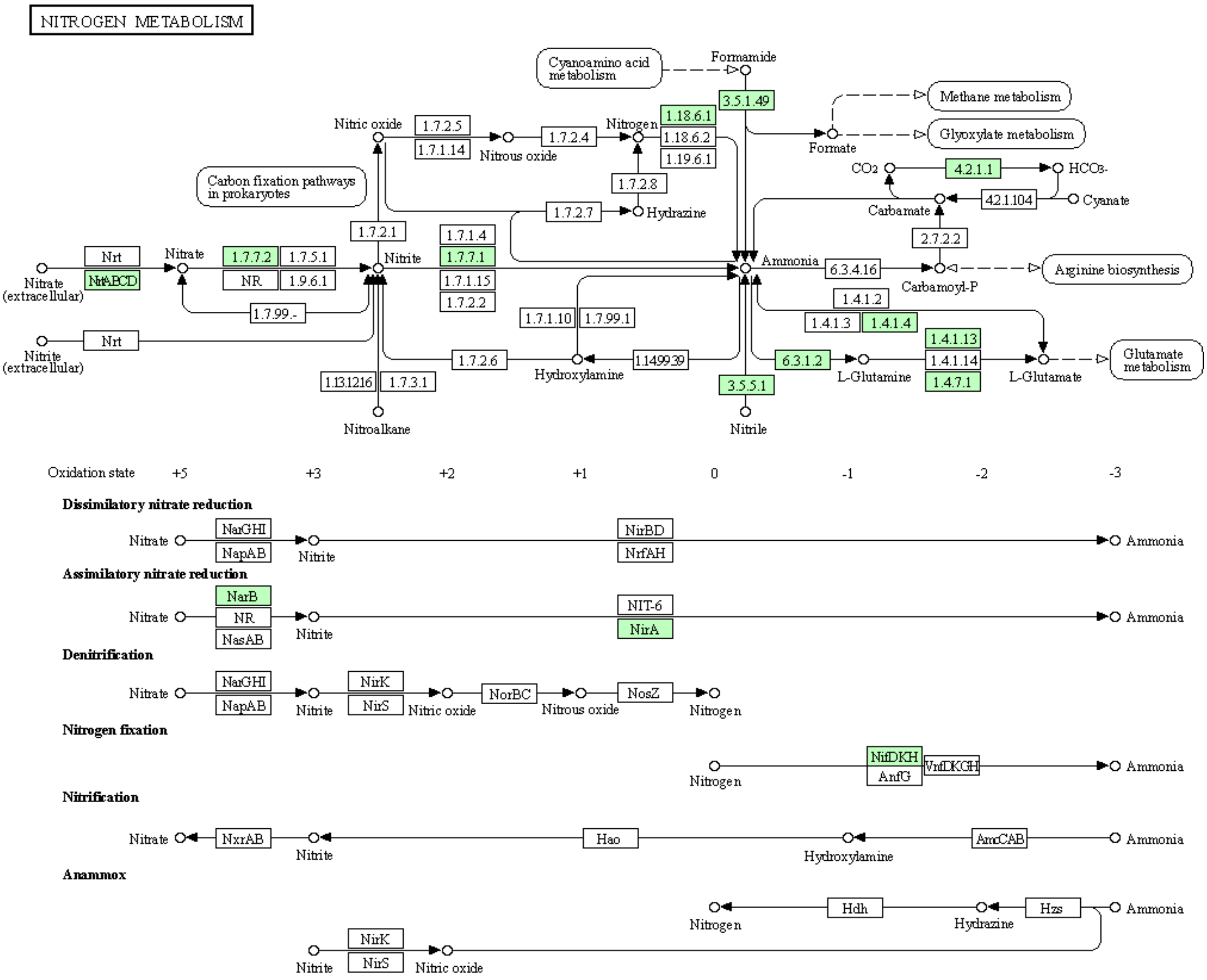

Figure.S2 KEGG pathway of nitrogen metabolism for strain B121

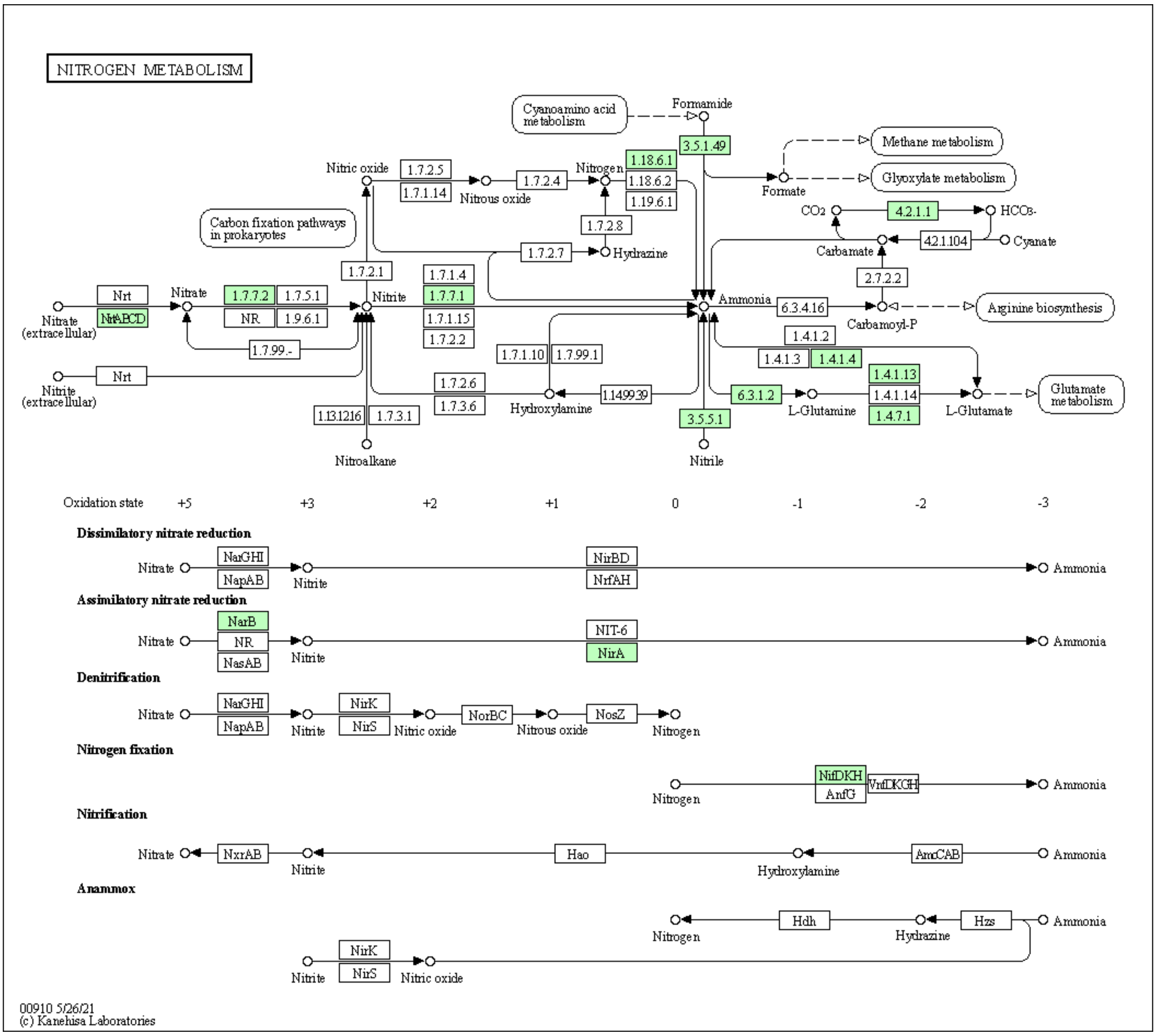

Figure.S3 KEGG pathway of nitrogen metabolism for strain A183

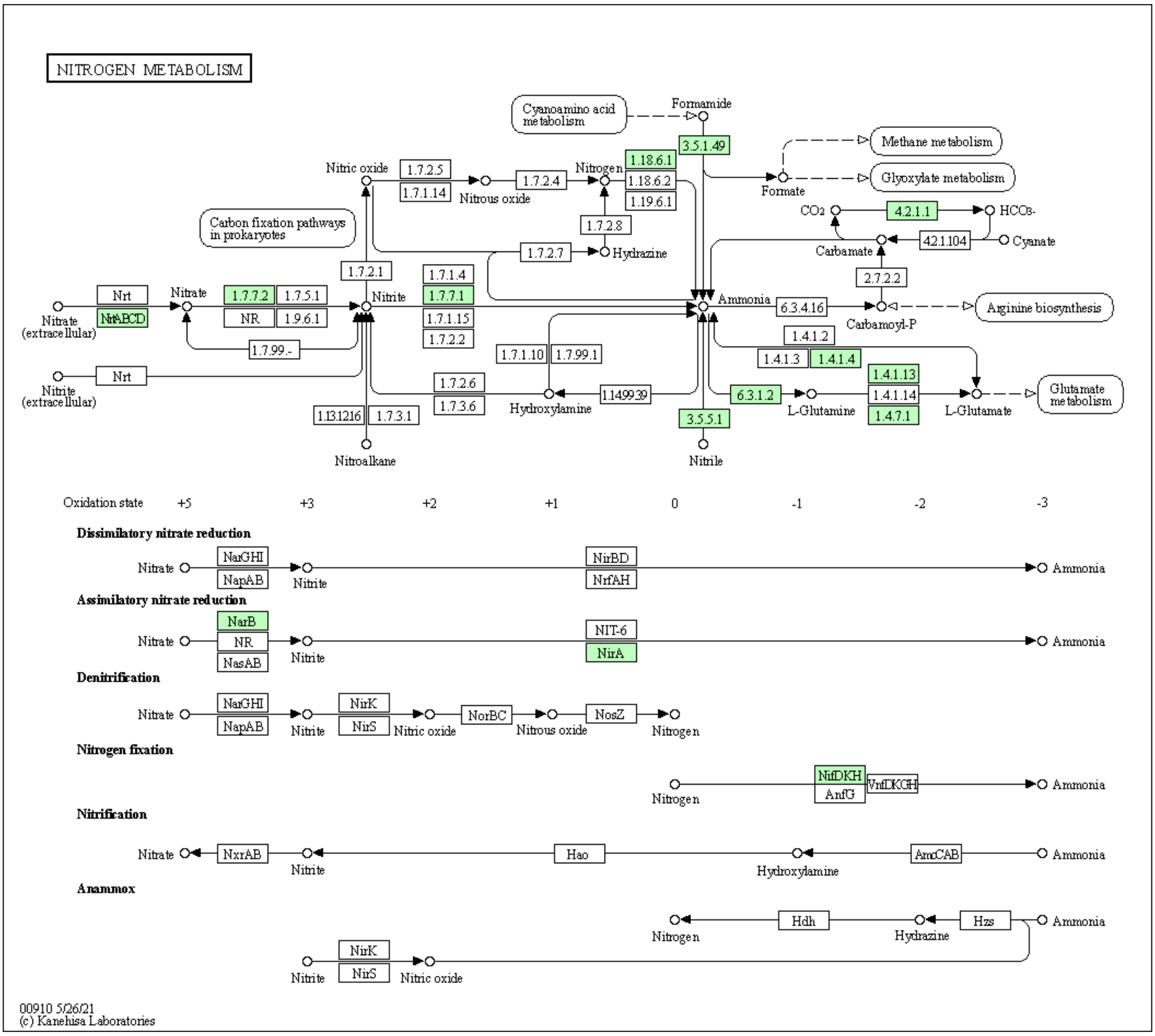

Figure.S4 KEGG pathway of nitrogen metabolism for strain *Thermoleptolyngbya* sp. O-77

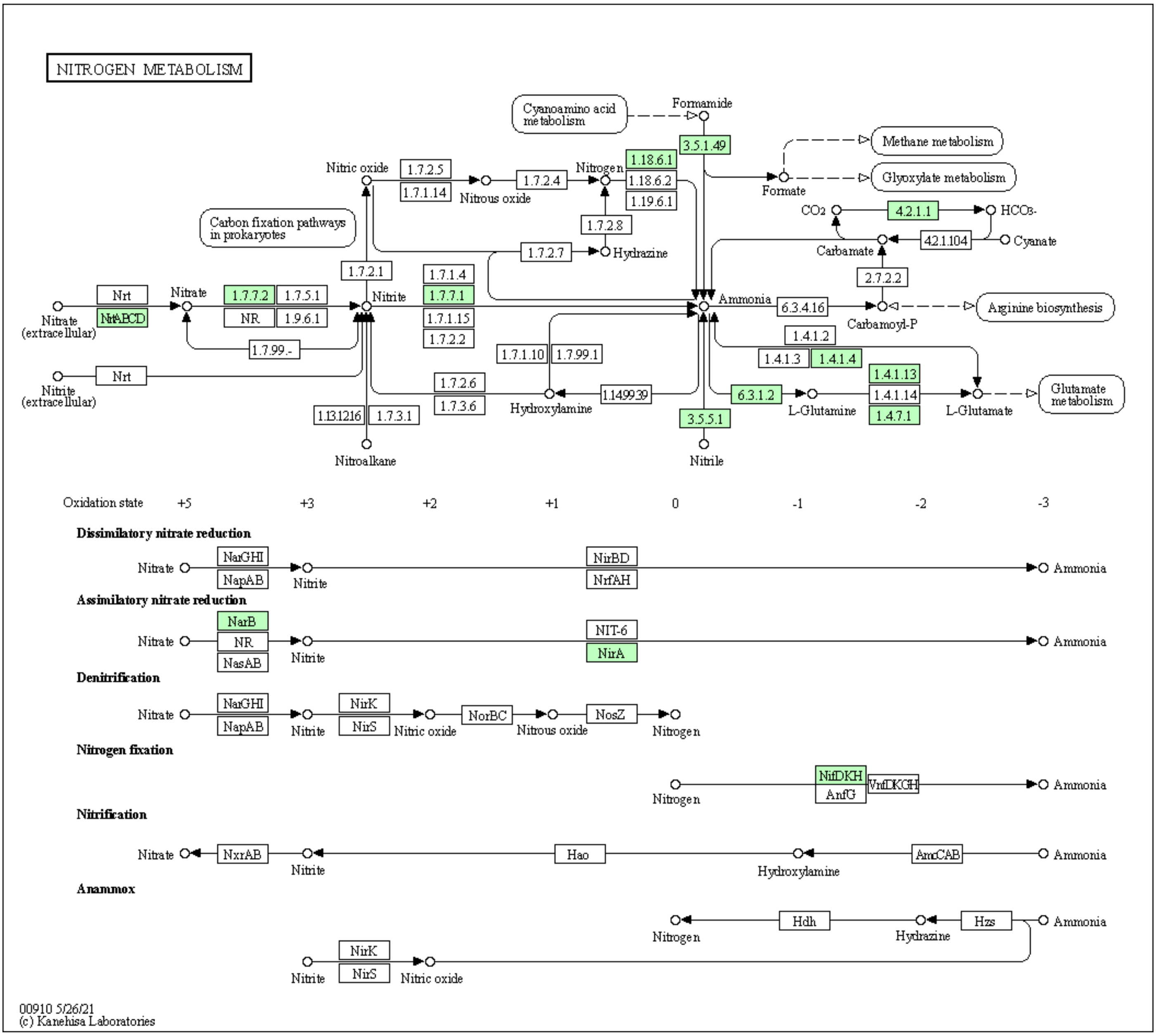

Figure.S5 KEGG pathway of nitrogen metabolism for strain *Thermoleptolyngbya* sp. C42_A2020
